# Subhepatic gallbladder: an extremely rare anatomical variant challenging the laparoscopic surgeon

**DOI:** 10.1093/jscr/rjag225

**Published:** 2026-04-04

**Authors:** Abdulaziz AlShehri, Hana AlZahrani, Anwar AlFadhel, Maram AlGarni, Sara Albawardi

**Affiliations:** Department of General Surgery, King Fahad Military Medical Complex (KFMMC), Old Abqaiq Road, Dhahran 34313, Eastern Province, Saudi Arabia; Department of General Surgery, King Fahad Military Medical Complex (KFMMC), Old Abqaiq Road, Dhahran 34313, Eastern Province, Saudi Arabia; Department of General Surgery, King Fahad Military Medical Complex (KFMMC), Old Abqaiq Road, Dhahran 34313, Eastern Province, Saudi Arabia; Department of General Surgery, King Fahad Military Medical Complex (KFMMC), Old Abqaiq Road, Dhahran 34313, Eastern Province, Saudi Arabia; Department of General Surgery, King Fahad Military Medical Complex (KFMMC), Old Abqaiq Road, Dhahran 34313, Eastern Province, Saudi Arabia

**Keywords:** subhepatic gallbladder, anatomical variation, laparoscopic cholecystectomy, MRCP, bile duct injury

## Abstract

Gallbladder anatomical variations are common, but positional anomalies such as subhepatic gallbladder are exceedingly rare. Recognition of these variants is crucial for preventing iatrogenic bile duct injury during laparoscopic cholecystectomy. A 35-year-old medically free male presented with a 3-day history of epigastric pain associated with nausea, vomiting, and dark urine. Laboratory investigations revealed elevated liver enzymes and hyperbilirubinemia. Ultrasonography showed gallstones with a dilated common bile duct. Magnetic resonance cholangiopancreatography (MRCP) demonstrated a subhepatic gallbladder containing multiple stones, an impacted neck stone, and a distal common bile duct (CBD) stone. Endoscopic retrograde cholangiopancreatography with sphincterotomy and stone extraction was performed, followed by stent placement. Laparoscopic cholecystectomy revealed the gallbladder in a subhepatic position surrounded by dense adhesions. Dissection of Calot’s triangle was carefully performed to identify and secure the cystic duct and artery. The procedure was completed successfully, and the patient’s postoperative recovery was uneventful. He was discharged home on postoperative Day 1 with improved liver function. Subhepatic gallbladder is a rare congenital anomaly that can mimic typical biliary disease but complicate surgical anatomy. Preoperative MRCP is invaluable in detecting such anomalies, guiding operative planning, and minimizing bile duct injury risk. Awareness of positional variations enhances surgical safety and decision-making. Preoperative imaging and intraoperative vigilance are essential when encountering atypical gallbladder anatomy. This case underscores the importance of anticipating anatomical variations to achieve safe and effective laparoscopic cholecystectomy.

## Introduction

The biliary system is composed of the intrahepatic ducts, the extrahepatic ducts, and the gallbladder [1]. Anatomically, the gallbladder occupies the right hypochondrial region, positioned in the gallbladder fossa on the visceral surface of the right hepatic lobe, interposed between hepatic segments IV B and V [2]. The biliary tree exhibits marked anatomical complexity, with structural variations reported in nearly 50% of all patients [3], encompassing variations in its number, size, morphology, and anatomical position [2]. Multiple atypical locations have been documented, including suprahepatic, intrahepatic, left-sided, retrocolic, retroduodenal, and retrorenal sites. To the best of our knowledge, only a single instance of a gallbladder positioned within the anterior abdominal wall has been reported [2]. Among these variations, the subhepatic gallbladder is a rare congenital anomaly of the gallbladder in which the organ is situated inferior to the hepatic margin, occupying a caudal position relative to the standard gallbladder fossa on the visceral surface of the right hepatic lobe. Patients harboring subhepatic gallbladder and presenting with symptomatic gallstones exhibit clinical manifestations that mirror those of individuals with a normally located gallbladder [1], making the clinical identification of this anomaly exceedingly challenging. For the surgeon, mastery of the biliary anatomy and its anatomical variations is critical, as it underpins the safe execution of laparoscopic cholecystectomy and the avoidance of iatrogenic injury to the biliary system [1]. This case report outlines a case of patient presenting with subhepatic gallbladder, pre-operative assessment and investigations was performed, and surgical management was applied, followed by observation through outpatient clinic.

## Case presentation

**Figure 1:**
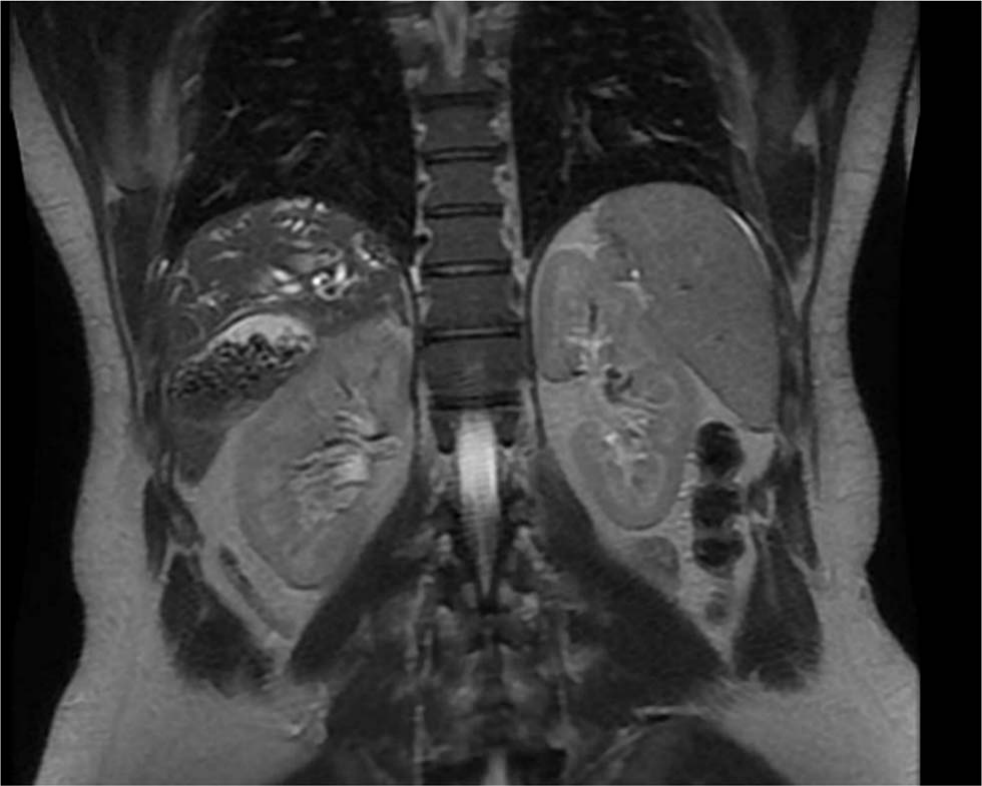
MRCP, coronal views, demonstrating an abnormally located subhepatic gallbladder positioned inferior to the liver margin.

**Figure 2:**
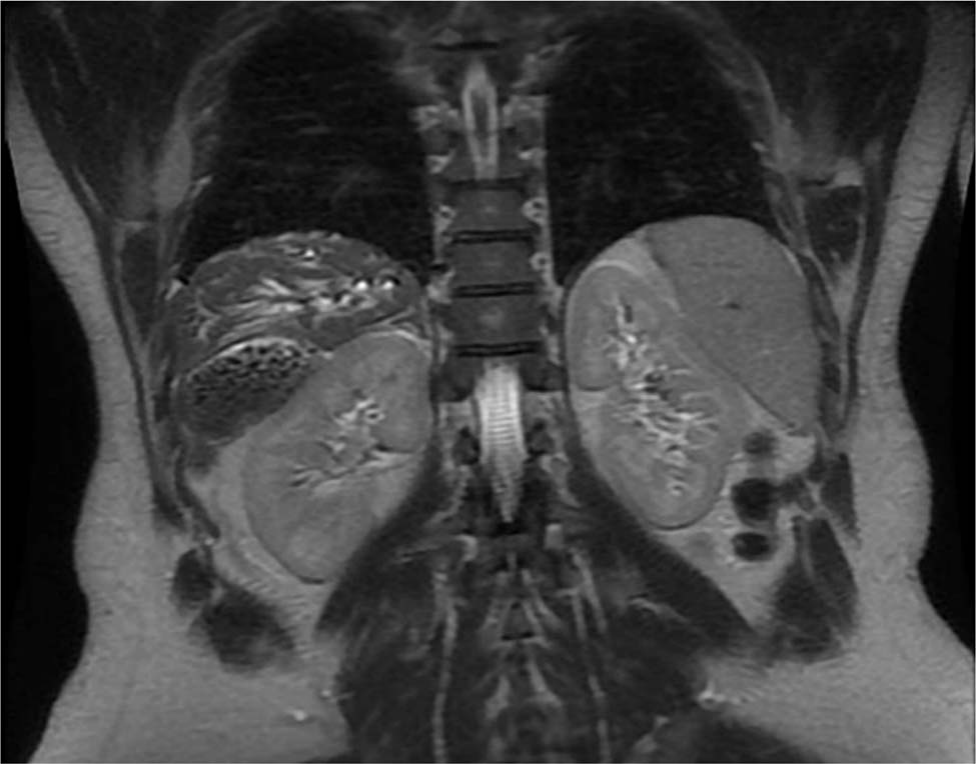
MRCP showing the gallbladder is markedly distended and contains multiple gallstones and sludge, with an impacted stone at the gallbladder neck. Associated dilatation of the common bile duct and intrahepatic biliary radicles is noted, consistent with obstructive biliary pathology.

A 35-year-old male, medically free, presented to the emergency department with a 3-day history of epigastric abdominal pain. The pain was gradual in onset, colicky, non-radiating, and aggravated by fatty meals. It was partially relieved by analgesia. He reported nausea with two episodes of vomiting and dark urine, but denied fever, rigors, or bowel habit changes. His past surgical history included laparoscopic sleeve gastrectomy performed 7 years earlier, with subsequent weight loss of 40 kg, and anal fistulotomy 1 year prior. On examination, the patient was alert, oriented, and afebrile. Conjunctival icterus was noted. Abdominal examination revealed right upper quadrant and epigastric tenderness with a positive Murphy’s sign. Laboratory investigations showed: hemoglobin 14.8 g/dl, WBC 5 × 10^9^/l, Alanine Aminotransferase (ALT) 341 U/l, Aspartate Aminotransferase (AST) 96 U/l, Alkaline Phosphatase (ALP) 250 U/l, Gamma-Glutamyl Transferase (GGT) 21 U/l, total bilirubin 121.9 μmol/l, and direct bilirubin 86.7 μmol/l. Ultrasound was done revealed multiple gallstones with gallbladder wall thickening, a dilated common bile duct (8 mm). Magnetic resonance cholangiopancreatography (MRCP) done and demonstrated an abnormally located subhepatic gallbladder, innumerable gallstones and sludge in a distended gallbladder with an 8 mm stone impacted at the gallbladder neck, moderate CBD dilation (1.1 cm), and a 6 × 4 mm distal CBD stone. (Fig. 1) Intrahepatic biliary radicles were dilated with a mildly irregular contour concerning for cholangitis. (Fig. 2).

**Figure 3:**
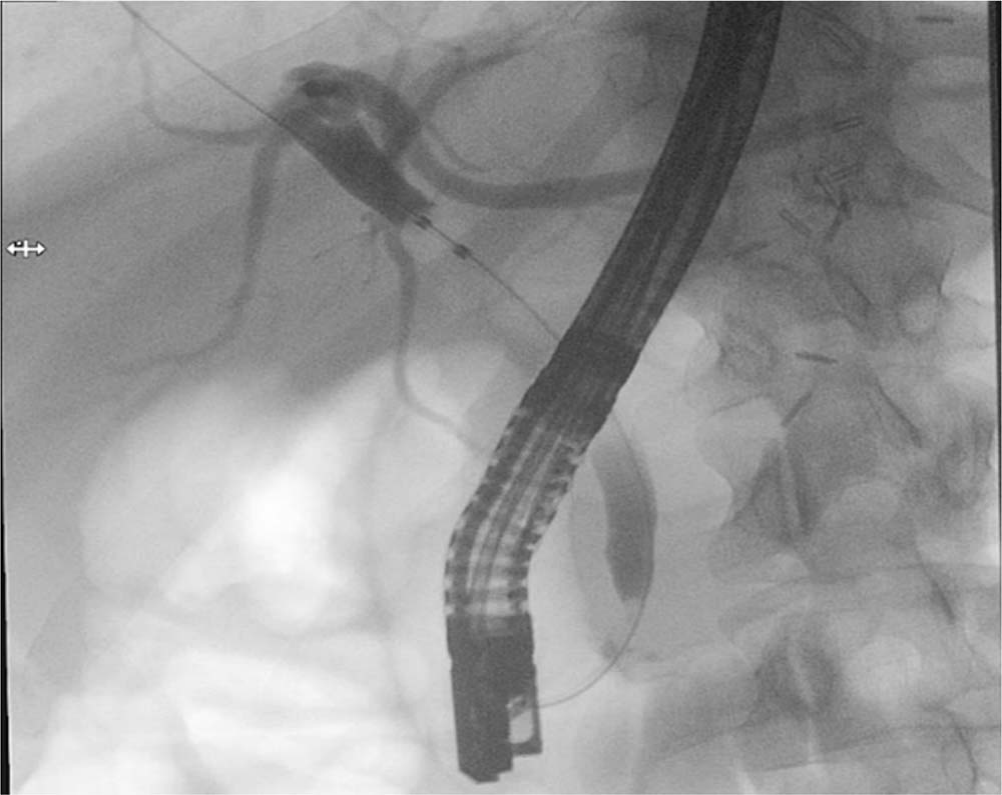
Endoscopic retrograde cholangiopancreatography fluoroscopic image showing contrast opacification of the biliary tree with a distal common bile duct filling defect, consistent with choledocholithiasis.

**Figure 4:**
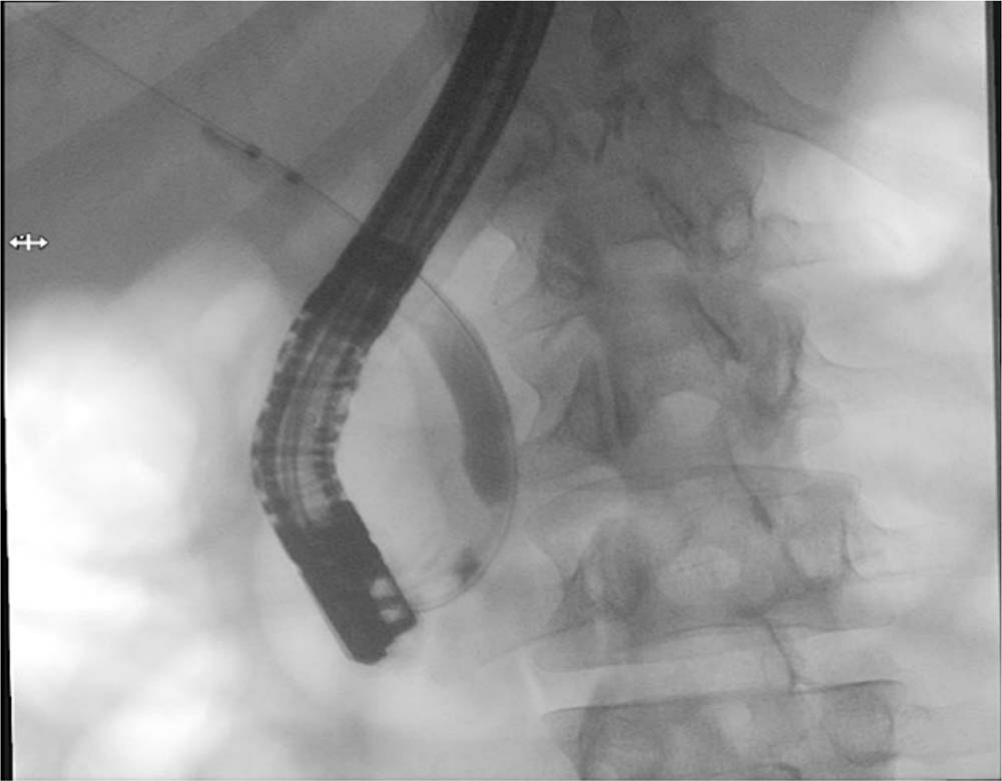
Therapeutic ERCP fluoroscopic image demonstrating successful biliary sphincterotomy, balloon stone extraction, and placement of a biliary stent within the common bile duct.

Patient subsequently performed endoscopic retrograde cholangiopancreatography with needle-knife cannulation. Cholangiogram confirmed a distal CBD filling defect. A sphincterotomy and balloon sweep were performed, successfully extracting stones, and an 8.5 Fr × 7 cm stent was deployed (Fig. 3, Fig. 4).

Eventually after full pre-operative assessment patient underwent laparoscopic cholecystectomy. Intraoperatively, the gallbladder was noted to be in an unusual subhepatic location extending to the right side, surrounded by dense adhesions and appearing inflamed and edematous. Retraction was technically challenging. Careful adhesiolysis and dissection of Calot’s triangle were performed to identify and skeletonize the cystic duct and artery, which were clipped and divided. The gallbladder was dissected from its bed using electrocautery, during which iatrogenic perforation with minor stone spillage occurred. The specimen was retrieved via the epigastric port. A drain was placed, and hemostasis was secured. The patient had an uneventful recovery.

In the postoperative period, Day 1 he was clinically well, vitally stable afebrile with mild tenderness at the surgical site. Laboratory values showed improving liver function: ALT 396 U/l, AST 253 U/l, ALP 132 U/l, total bilirubin 32 μmol/l, and direct bilirubin 21 μmol/l. The drain output was minimal and serous. Patient assessed again on the same day, he is doing fine, drain removed and patient discharged home safely with follow up in outpatient clinic.

## Discussion

This case describes a 35-year-old male with a subhepatic gallbladder—an uncommon congenital anomaly—presenting with acute calculous cholecystitis and choledocholithiasis. Anatomical variations of the gallbladder are relatively frequent; however, positional anomalies such as subhepatic or ectopic gallbladders are rare, with an incidence of less than 1%. They are often discovered incidentally during imaging or surgery because clinical symptoms mimic typical biliary colic [4–6].

In our patient, preoperative MRCP accurately identified the subhepatic location, which allowed for careful operative planning. Most previously reported cases describe the anomaly being detected intraoperatively, increasing the risk of iatrogenic injury.

Awareness of gallbladder positional variations is crucial for surgeons to prevent bile duct injury, particularly during laparoscopic cholecystectomy where altered anatomy increases the risk of iatrogenic complications. Anomalies such as subhepatic, intrahepatic, or left-sided gallbladders are uncommon but clinically significant, as they can alter the expected orientation of Calot’s triangle and biliary structures, making dissection technically demanding [6, 7]. Failure to recognize these variations preoperatively may lead to misidentification of biliary anatomy and subsequent bile duct injury or conversion to open surgery [8].

From an embryological perspective, positional anomalies of the gallbladder are thought to result from abnormal migration of the hepatic diverticulum during early fetal development. The gallbladder and bile ducts originate from the caudal portion of the hepatic diverticulum, and failure of proper rotation or migration can result in ectopic positioning such as subhepatic, intrahepatic, retrohepatic, or even left-sided locations. Understanding these developmental mechanisms not only aids in interpreting unusual imaging findings but also reinforces the importance of anatomical awareness during hepatobiliary surgery.

Preoperative imaging plays a pivotal role in diagnosis. When the gallbladder is not visualized in its usual fossa on ultrasonography, further evaluation with MRCP is recommended to delineate the biliary anatomy and detect associated anomalies [9, 10]. MRCP provides a noninvasive, detailed assessment of the biliary and pancreatic ducts, allowing better surgical planning and risk reduction [11, 12].

Intraoperatively, meticulous dissection and the application of the “critical view of safety” technique are essential when dealing with atypical gallbladder anatomy. Surgeons should identify only two structures entering the gallbladder—the cystic duct and cystic artery—before transection. When visualization of Calot’s triangle is unclear, adjuncts such as intraoperative cholangiography, indocyanine green fluorescence imaging, or conversion to open cholecystectomy may be warranted to minimize the risk of bile duct injury. A methodical and patient approach, combined with preoperative imaging, significantly reduces intraoperative uncertainty and enhances patient safety.

Although our patient experienced a favorable postoperative recovery, continued long-term follow-up remains essential to identify delayed biliary complications such as stricture formation, retained stones, or stent-related issues.

This case reinforces the importance of maintaining a high index of suspicion for positional gallbladder anomalies, careful preoperative assessment, and intraoperative vigilance to ensure patient safety and optimal outcomes [13].

## Conclusion

Subhepatic gallbladder is an exceptionally rare congenital positional anomaly that can pose significant diagnostic and operative challenges. Because clinical manifestations often mimic those of a normally located gallbladder, preoperative imaging—particularly MRCP—is essential for accurate localization and surgical planning. Surgeons must maintain a high index of suspicion when imaging findings are atypical or when the gallbladder is not visualized in its usual fossa. Meticulous operative technique, adherence to the critical view of safety, and readiness to employ adjunctive imaging or convert to open surgery when anatomy is unclear are key to preventing bile duct injury. This case highlights the indispensable role of anatomical awareness, careful preoperative assessment, and intraoperative vigilance in ensuring safe and successful outcomes in patients with biliary anatomical variations.
